# Care opportunities for premature infants: home visits and telephone support*

**DOI:** 10.1590/1518-8345.3520.3308

**Published:** 2020-07-01

**Authors:** Rosane Meire Munhak da Silva, Adriana Zilly, Eliana Roldão dos Santos Nonose, Luciana Mara Monti Fonseca, Débora Falleiros de Mello

**Affiliations:** 1 Universidade Estadual do Oeste do Paraná, Campus de Foz do Iguaçu, Foz do Iguaçu, PR, Brazil.; 2 Scholarship holder at the Fundação Araucária - Cp 16/2017, Foz do Iguaçu, PR, Brazil.; 3 Universidade Estadual do Oeste do Paraná, Hospital Universitário do Oeste do Paraná, Cascavel, PR, Brazil.; 4 Universidade de São Paulo, Escola de Enfermagem de Ribeirão Preto, PAHO/WHO Collaborating Centre at the Nursing Research Development, Ribeirão Preto, SP, Brazil.

**Keywords:** Infant, Premature, Child, House Calls, Telemedicine, Health Promotion, Primary Health Care, Recém-Nascido Prematuro, Criança, Visita Domiciliar, Telemedicina, Promoção da Saúde, Atenção, Recien Nacido Prematuro, Niño, Visita Domiciliaria, Telemedicina, Promoción de la Salud, Atención Primaria de Salud

## Abstract

**Objective:**

to analyze opportunities for orientations to promote the care of premature infants during home visits and telephone support.

**Method:**

a qualitative study from the perspective of philosophical hermeneutics conducted with 18 mothers of premature infants discharged from hospital. Hospital contact and interviews were carried out, 15 and 45 days after discharge and at the infants’ six months of life, with data analysis by interpretation of meanings from 25 home visits and 56 telephone support contacts.

**Results:**

the following two thematic units emerged: Needs for contact and guidance: the place for home visits and opportunities for resolving doubts by telephone support, indicating aspects that suggest weakening child health, discontinuity in follow-up and vulnerability in specialized follow-ups. Home visits and telephone support favored the concern of health needs, doubts about basic care and problem solving, as ways to prevent damage and promote child health.

**Conclusion:**

home visits and telephone support emerge as collaborative practices of care and detection of latent conditions, which can be reduced or interrupted with prompt return of guidance, suggesting opportune strategies to increase follow-up, linkage and access to the health services.

## Abstract (pt)

**Objetivo:**

analisar oportunidades de orientações para promoção do cuidado de crianças prematuras em visita domiciliar e suporte telefônico.

**Método:**

estudo qualitativo na perspectiva da hermenêutica filosófica com 18 mães de crianças prematuras egressas de unidade hospitalar. Realizou-se contato hospitalar e em seguida entrevistas, aos 15 e 45 dias após a alta e aos seis meses de idade da criança, com análise de dados por interpretação de sentidos, a partir de 25 visitas domiciliares e 56 suportes telefônicos.

**Resultados:**

duas unidades temáticas emergiram: Necessidades de contato e orientações: o lugar das visitas domiciliares e Oportunidades de resolução de dúvidas por suporte telefônico, indicando aspectos que sugerem fragilizar a saúde infantil, descontinuidade no seguimento e vulnerabilidade nos acompanhamentos especializados. Visita domiciliar e suporte telefônico favoreceram a apreensão de necessidades de saúde, dúvidas de cuidados básicos e resolução de problemas, como formas para prevenir danos e promover saúde infantil.

**Conclusão:**

visita domiciliar e suporte telefônico despontam como práticas colaborativas de cuidados e detecção de condições latentes, que podem ser reduzidas ou interrompidas com prontidão de retorno de orientações, sugerindo estratégias oportunas para incrementar seguimento, vinculação e acesso aos serviços de saúde.

## Abstract (es)

**Objetivo:**

analizar las posibles directrices a seguir para fomentar el cuidado de bebés prematuros, mediante visitas a domicilio y asistencia telefónica.

**Método:**

estudio cualitativo desde una perspectiva hermenéutica filosófica, realizado con 18 madres de bebés prematuros dados de alta en una unidad hospitalaria. Se llevaron a cabo contactos en el hospital seguidos de entrevistas, 15 y 45 días después del alta médica, así como a los seis meses de vida de los bebés, analizando los datos por interpretación de sentidos, a partir de 25 visitas a domicilio y 56 contactos de asistencia telefónica.

**Resultados:**

surgieron dos unidades temáticas: Necesidades de contacto y directrices: el lugar de las visitas a domicilio y la Posibilidad de resolver dudas a través de la asistencia telefónica, poniendo de manifiesto aspectos que indican la fragilidad de la salud infantil, la falta de continuidad en el seguimiento y la vulnerabilidad en los seguimientos especializados. Las visitas a domicilio y la teleasistencia ayudaron a identificar las necesidades sanitarias y las dudas sobre cuidados básicos. También fueron útiles para resolver problemas, como formas para prevenir dolencias y para promover la salud infantil.

**Conclusión:**

las visitas a domicilio y la asistencia telefónica se destacan como buenas prácticas colaborativas en lo que se refiere a la atención y detección de patologías latentes, pudiendo estas disminuir o desaparecer, como consecuencia de la inmediatez en las respuestas. Esto nos lleva a que se sugieran como estrategias oportunas para mejorar el seguimiento, la vinculación y el acceso a los servicios de salud.

## Introduction

Premature births add up to approximately 15 million annually worldwide and are a public health problem because they are a risk factor for infant morbidity and mortality^(^ 1 ^)^. The literature points out that, after the hospitalization period, premature infants have health complications, and related to neurodevelopment in the first year of life and in the long term^(^ 1 ^-^ 2 ^)^. Thus, home care is a challenge for families, with different health care needs^(^ 2 ^)^.

Access to health practices and continuity of care become essential, since premature infants require individual and singular care, due to the vulnerabilities and needs for continuous care to ensure health and development as well parental caregivers that require preparation and support to exercise care^(^ 3 ^)^. In this way, the performance of the health professionals is relevant for interventions with the families^(^ 4 ^)^. For this reason, they should use approaches to monitor premature infants in order to meet their needs and those of their families, considering that the mothers may be inexperienced. Evidence shows the importance of approaches in family environments through Home Visits (HVs) and/or telephone support, increasing health, survival, and development^(^ 5 ^)^.

In child health, the HV is addressed to assess the mother-child interaction and child care^(^ 6 ^)^, to identify and intervene in vulnerable situations, to collaborate to improve the trajectories of women, children and families, with a positive impact on maternal and child health^(^ 7 ^)^, in the development of the bond, with increased breastfeeding rates, decreased smoking and mothers’ return to work or school^(^ 8 ^)^. However, investments to structure the teams of primary care services and to organize health actions in households are limited in Brazil^(^ 9 ^)^.

Telephone support, reported as a way to improve health care access and efficiency, has good acceptability for families with children in vulnerable situations^(^ 10 ^)^; however, it is a little explored practice in Brazil. The use of technology, such as mobile phones, is accessible to the population and services, in developed countries or not^(^ 11 ^)^.

In view of the relevance of the aforementioned strategies, the study aimed to analyze opportunities for guidance to promote the care of premature infant in HV and support through telephone.

## Method

A qualitative study from the perspective of philosophical hermeneutics^(^ 12 ^)^, as a comprehensive-interpretative movement that permeates human experience, rooted in processes and everyday acts of understanding and dialog.

18 mothers were included in the research, by intentional sampling, who met the inclusion criteria: age ≥ 18 years old, with infants born with a gestational age under 37 weeks, hospitalized for at least five days, and residing in Foz do Iguaçu-PR-Brazil. The exclusion criteria were the following: mothers diagnosed with mental health problems recorded in medical records, communication difficulties due to ethnic diversity considering that the municipality belongs to the triple border, next to Ciudad del Este (Paraguay) and Puerto Iguazú (Argentina), and impossibility to perform the HV due to the lack of location of the address and/or absence of the mother after three attempts.

The Neonatal Intensive Care Unit (NICU) studied is the only one of high complexity in the region, responsible for caring for sick newborns in the municipalities belonging to the Foz do Iguaçu regional health area, and also for serving a population beyond the programmed, considering that the city is touristic, added to the health demands of neighboring countries, caused by the care vulnerabilities of the municipalities in this border strip^(^ 13 ^)^. There is no specific follow-up service for post-discharge pre-term infants, and this monitoring is carried out by the Center for Child Nutrition in the city, which has a pediatrician, a nurse and a nutritionist to care for premature or low weight infants.

Being a qualitative research, the search and inclusion of participants was completed when the results generated meanings to understand the phenomena in depth, richness and complexity^(^ 14 ^)^.

Between July 2017 and April 2018, data collection was organized in four stages: a meeting at the hospital to establish the first contact, considering that the interviewer did not know the mothers, a HV and two telephone support contacts, conducted by the lead author, who has experience in neonatal nursing and qualitative research. During the data collection there were new contacts, in person and via telephone, driven by maternal doubts/difficulties to care at home. Thus, some stages occurred more than once, totaling 25 HVs and 56 telephone support contacts (25 phone calls and 31 instant text messages).

Initially, at the hospital and in a private location, each participant was contacted and selected based on the inclusion criteria, while the child remained hospitalized, with presentation of the objectives and of the free and informed consent term, with a mean duration of 15 minutes. One mother refused, since a twin son remained hospitalized. 15 days after the baby was discharged from the hospital, the HV was performed at the mother’s own home to proceed with the interview, lasting 40 minutes. On some occasions, other family members were present (father, grandparents, and siblings), but they did not participate in the interviews. 45 days after discharge and at six months of the child’s chronological age, the mothers were contacted via phone calls and/or instant text messages.

The technique chosen for data collection in the HV was the interview, conducted based on the following guiding question: “Tell me how your daily care with your child has been?”, recorded on audio and later transcribed. For telephone contacts, the dialog and the search for information was conducted by a semi-structured instrument that portrayed potentialities and difficulties for home care, recorded directly by the researcher and then read for the participant’s consent. For text messages, phone calls or search for HV, driven by the mothers, a defined script was not followed, they occurred according to the demand of each participant, and these moments were recorded in the Field Diary. When there was an intervention with guidance in the face of maternal difficulties, they were also recorded in the Field Diary.

Data analysis was guided by the interpretation of meanings, with repeated readings of the empirical material, with a broader view of the whole (care of the premature, HV, and telephone support) and its particularities, in search of understanding of meanings, reinterpretation and explanation of the contents^(^ 15 ^)^ without using data analysis software.

The context of the HV and of telephone contacts generated several elements concerning the care of the premature infant, such as: maternal concerns for home care and the child’s development; guidance and support for care; ignorance of health follow-up flows; family environment and security; opportunities for guidance and resolution of doubts; and promoting child development. From such subthemes significant dimensions emerged and were grouped into thematic units: Needs for contact and guidance: the place for home visits, and Opportunities for resolving doubts by means of telephone support.

To maintain anonymity, the participants were identified by the letter P and numbering (P1 to P18), and the time of the intervention by the acronym HV (Home Visit, 15 days after discharge), Phone Call I (45 days after discharge) or Phone Call II (at six months of age). The Field Diary was identified as Diary-HV, Diary-1^st^Phone Call or Diary-1^st^ *WhatsApp*.

The investigation was approved by the Research Ethics Committee, complying with the research rules involving human beings.

## Results

The participants have a mean age of 28 years old, most of them with a paid profession, living with a partner and having undergone surgical delivery due to health problems. Their children were born with a mean gestational age of 32 weeks, a mean birth weight of 1715 g and a mean hospitalization time of 26 days. Most receive assistance from the public health service and, for monitoring their child’s health, they seek the Child Nutrition Center in Foz do Iguaçu.

### Contact needs and guidance: the place for the home visits

Maternal reports express concerns for home care, with emphasis on increased attention to premature infants. *The nursing staff is aware that the baby doesn’t break, but we think they’re ridiculously small. It’s all new for us. It’s different to bathe a three kilogram baby. Care must be doubled. And not knowing, because you’ve never done it, to do it at home, without professional assistance, it’s quite different*(P13, HV); *In the first nights, I wouldn’t wait him to wake up, to start crying. He can lower glucose and we don’t even notice. This care of the time to breastfeed is because he’s premature and underweight*(P17, HV).

The HV made it possible to apprehend maternal concerns that can weaken health surveillance and potentiate insecurity to care at home. *In the HV the mother had doubts about the position of the daughter to offer the bottle feeding. The interviewer demonstrated the position and she was guided to administer it after breast milk was offered*(P1, Diary-HV); *The mother expressed concern about a health problem of another family member, which left her weakened, making her insecure to take care of her child*(P6, Diary-HV).

Contacts through HVs provided opportunities to offer guidance in the face of the situations listed by mothers, with identification of the circumstances that triggered nursing intervention in the health care network. *The child wasn’t undergoing outpatient follow-up and the mother had doubts. Oriented on childcare and made contact with the family health unit regarding the active search. The child developed hyperthermia and received medical attention, and an antibiotic was prescribed. The mother had doubts about how to get the antibiotic without having to buy it. Contact was made with the pharmacy of the health unit and the family started treatment*(P9, Diary-HV); *The researcher advised on practices that facilitate the bath of the premature, ways to organize the environment and hold the baby*(P13, Diary-HV); *The researcher encouraged the mother to maintain care with feeding times, seeking to respect the intervals established by the baby, avoiding an extended time that puts her health at risk*(P17, Diary-HV).

In addition to the need for specialized monitoring, there is a certain lack of maternal knowledge about the organization and flow of care. *The hospital pediatrician gave me referral for surgery* [hypospadias correction] *to take to the health center, check in, I don’t know how it works*(P6, HV); *The mother had doubts related to referrals with specialists received at the hospital, blood tests* [for follow-up of congenital syphilis treatment] *and how to acquire the results of imaging tests performed at the hospital. Orientation and intermediation for consultations and examinations in the health unit and hospital*(P8, Diary-HV).

At these times, intermediation from the HV was recognized as an important guiding tool, to guide the search for health care.

There are other aspects that were identified from the HV, which express a relationship with the environment and family security. *There was a maternal concern in relation to the family’s financial conditions, she has three children and finds it difficult to obtain reclusion aid* [partner in prison]. *The researcher instructed to seek social assistance from the health district to which she belongs to support her in this process*(P7, Diary-HV); *The environment around the family residence presents objects liable to accumulate dirt, insects and rodents, placing collective health in a vulnerable situation. The health situation of the maternal grandmother, who has a mental health problem* [psychological disorder due to compulsive accumulation]*, known to the professionals of the health unit, was identified. Observation of police report of the Women’s Special Police Office, carried out by the participant, referring to marital conflicts that involved physical and verbal aggression between the couple. The researcher went to the health unit, noting that the situation of violence was known to the professionals, and the participant herself had already declined to the police report. The unit is not accompanying the family in HV*(P8, Diary-HV).

The performance of HV in the present study showed the potential for greater bonding and dialog with mothers, highlighting the needs for communication with health professionals, in addition to the scheduled consultations. The home meetings expressed the real needs, difficulties and concerns for the care and attention to the health of the premature infant, in addition to environmental and socio-emotional aspects.

### Telephone support troubleshooting opportunities

The mothers’ reports through telephone calls and text message contacts point to aspects of daily care and suggest opportunities for resolving doubts. *Contact made by the mother seeking guidance on diaper dermatitis*(P1, Diary-1st Phone call); *New contacts were made for guidance and resolution of dermatitis*(P1, Diary-2nd, 3rd Phone Calls); *Contact made by the mother to seek guidance on how to proceed with the daughter who was feverish and with nasal congestion. Oriented to perform baths and compresses, to ventilate the environment, to elevate the cradle’s head and to hydrate nostrils with physiological solution, reinforcing the importance of seeking care in the family health unit*(P14, Diary-1st WhatsApp).

The identification of aspects that need guidance and the establishment of contacts between mothers and professionals, in addition to the meetings previously scheduled in routine consultations, show that different doubts and concerns arise in daily life, related to health, development and basic care of child.

The mothers recognize the importance of the contact with the health professionals, especially to support them in addressing the needs of prematurity. *I think the contact with professionals is valid, it’s a bond that parents need, in all segments for their children. Each professional always leaves a recommendation or learning*(P5, 2nd Phone Call); *I believe I need to talk to her. Just yesterday I bought some books to start telling stories. She needs games. If I turn her face down, she doesn’t move or turns around, she gets angry because she can’t and cries. She doesn’t answer by name, but for other things she’s curious, she looks at everything, any noise she seeks. Even breastfeeding, if someone speaks, she stops breastfeeding to look for*(P18, 2nd Phone Call); *Contact was made by the mother to seek information about the daughter’s development and she was instructed on the need to perform appropriate stimuli, examples were given and reinforcement on follow-up by specialized professionals, according to medical recommendation*(P5, Diary-1st WhatsApp).

Telephone contact around the sixth month of the child’s chronological age enabled the apprehension of the development details, with reinforcement of appropriate stimuli and detection of situations of improvement in home care. Other reports show that the mothers considered telephone support viable as a communication and support network. *Spontaneous contacts made via WhatsApp by the mother to clarify doubts about the appearance of the child’s feces, how to transport the daughter and related to exposure to other environments*(P18, Diary-1st, 2nd, 3rd WhatsApp); *I think the contact is good because, often, something happens in the middle of the night or weekend and the health services are not available*(P13, 2nd Phone Call); *For me, both by phone and personally it helps a lot* (P16, 2nd Phone Call); *Having someone you can trust to answer questions, have support when you need it, that helps answering questions about the premature infant’s development, mainly because oxygen was lacking, because he had a low Apgar at birth*(P3, 2nd Phone Call); *I think these contacts help, because there are many doubts about many things. All of this could be solved in just one phone call*(P17, 2nd Phone Call).

The mothers emphasize the benefits of professional support for home care as an agile way of solving doubts, and reinforce that support is important both in person and by phone, suggesting new ways of bonding with the health professionals.

## Discussion

Through dialog and understanding, it was possible to perceive that the mothers had concerns about the health of their children because they were born premature and for the need of home care. The needs for contact, guidance and resolution of doubts indicated aspects that suggest weakening child health, discontinuity in the follow-up of growth and development in primary care services and vulnerability in specialized follow-ups. The strategies of HV and telephone support favored the perception of health needs and the daily doubts of basic care and provided the resolution of problems, as ways to prevent damage and promote child health in a situation of prematurity.

The HV and telephone support triggered the readiness to return to the guidelines, suggesting that they are opportune strategies to solve the problems identified by the mothers, as well as seeking health care when the child needs it.

Caring for premature infants at home requires careful assessment, considering family characteristics, emotional state of caregivers, stress levels, adaptation strategies, forms of home organization and the need for personal and professional support^(^ 16 ^)^. Several studies show that the combination of these factors results in support needs at home and strategies to meet involve integrated practices and services and assistance for urgencies and emergencies, including HV and telephone support carried out by qualified professionals^(^ 16 ^-^ 17 ^)^.

Although regulated, home care in the Unified Health System still has a character of complementary action in health networks and there is a deficit of home services on the national setting, when compared to other countries, such as Canada and the United States^(^ 9 ^)^.

The child’s daily events at home were pointed out by the mothers as deserving attention. The literature addresses that premature babies sleep more, breastfeed slowly and often do not even wake up to breastfeed^(^ 18 ^)^, it is important to keep track of feeding times to avoid hypoglycemia, brain damage, coma, and death^(^ 19 ^)^. Premature infants are smaller and are more likely to have hypotonia compared to full-term babies, and handling in a hygienic situation requires skills and freedom from fears about difficulties in body management and insecurity for the care of a small child^(^ 20 ^)^. In order for parental caregivers to face these new care situations, it is essential that the health team explain in detail each orientation before hospital discharge, in a welcoming manner, following the procedures in their execution and checking if, in fact, they are skilled and confident to carry them out^(^ 3 ^-^ 4 ^,^ 20 ^)^. The experience of prematurity leads to contact with experiences that are beyond the knowledge and daily life of mothers and families^(^ 21 ^)^, which requires continuity of support.

The HV, considered an ancient care technology, brings favorable results and is important for the family, especially in vulnerable situations such as prematurity and those that recently come out of NICU^(^ 22 ^)^. The HV establishes a closer relation with the family environment, routines, culture and attitudes towards health care^(^ 17 ^,^ 22 ^)^. These aspects show to be essential to ensure proper child development.

Premature children may have complex needs at home, with more difficulties for caregivers and possible errors in relation to food, medication and use of health equipment or services^(^ 22 ^-^ 23 ^)^. The active presence of health professionals at the homes can anticipate the identification of errors and difficulties of caregivers, helping them to apply effective practices to enhance care for their children^(^ 23 ^)^.

In addition to the contribution of HV to the care of premature babies, telephone support has also been recognized as a way to improve access and efficiency in health care^(^ 24 ^)^, the similarity of the results of the present research that suggest the relevance of these strategies for the promotion of care for premature infants at home.

A number of research studies point out that the uses of new technologies need adaptations to increase health care, and some promising results have been recorded, both to follow the child’s development and health conditions and to reduce the demand for hospital services^(^ 11 ^,^ 25 ^)^.

This study showed that doubts arise in home care, the dialogical relationship with mothers was increased by the communication of telephone contact and text messages, allowing for several clarifications. Therefore, strategies capable of preventing diseases are important for global development and in unexpected situations that can harm the child’s health^(^ 26 ^)^.

Another aspect to highlight refers to the maternal recognition of the needs of the premature’s development and its encouragement at home. The telephone approach included guidelines that seek to promote child development, such as talking, singing, giving objects to hold, among others. Offered in an appropriate way, these stimuli are important in care, whether for changing diapers or for specialized professionals^(^ 27 ^)^, since early childhood needs care, affection and interaction, to outline the way for the child to explore its potential and become a healthy and balanced adult^(^ 28 ^)^.

It is important to highlight the relevance of the family environment and the possibilities that HV and telephone support can offer. This study made it possible to analyze maternal needs, including the care of premature children and the family and social context. Different aspects can compromise care and safety at home and its surroundings, as families in situations of vulnerability, such as those presented, are less prepared to take care of their child at home and need an effective transition process^(^ 28 ^-^ 29 ^)^.

On these occasions, having professional support allowed the mothers to express their concerns, and the dialog and guidelines drove possible solutions, suggesting new dimensions for safe practices for the development of premature infants at home. The experience of the meeting and the search for broadening horizons guided the understanding and interpretation in this study, mediated by the dialogical movement and its potential^(^ 12 ^)^. Thus, in order to implement home care networks for premature infants, articulated interventions are essential, with programs and protocols for follow-up and continuous monitoring, ensuring longitudinality of care^(^ 4 ^,^ 16 ^,^ 21 ^)^. Although the families received support from the Child Nutrition Center, it is not linked to the Family Health Strategy; therefore, they do not perform HVs, showing ruptures in the system that leave these mothers helpless in the home.

These results can contribute to direct and encourage the health professionals to support parental caregivers with a view to improving the care of premature babies at home and strengthening parenting skills, revitalizing the dialog and ensuring child and family-centered approaches, focused on attention to needs and singularities, promotion of healthy growth and development, and prevention of diseases and infant mortality.

As limitations, we must signal the centrality of maternal reports and follow-up in the first post-discharge months, considering that complications and/or difficulties to take care at home may arise beyond the child’s six months of age, and new contacts by HVs or telephone support contacts will be important to support families and contribute to child development.

## Conclusion

The opportunities for guidance in HV and telephone support proved to be relevant for promoting the care of premature infant, particularly in the face of fragilities for child health, maternal doubts about basic home care, situations of discontinuity in the follow-up of growth and development and vulnerabilities specialized follow-ups. The HV and telephone support emerge as collaborative practices of preventive health care and detection of latent conditions, which can be reduced or interrupted.

Telephone contact and text messages may not have the same effect as a dialogical and shared relationship with mothers, which has the potential to favor exchanges of knowledge and practices essential to the care of premature infants, requiring more effective contact, communication and dialog and which contribute to expanding care between one person and another. However, communication by telephone conversations and written messages is favorable for resolving doubts, reinforcing teachings and facilitating referrals.

It is important to highlight that weaknesses in the health care networks and in the mechanisms for monitoring premature infants and those discharged from neonatal units, the absence of HVs and gaps for integration and continuity of care generate more vulnerabilities for children and their families who are already in fragile physical and psychosocial circumstances.

It is worth pointing out the relevance of the involvement of the health professionals to increase these action strategies, seeking ways to intervene and optimize the use of tools in the management of care in primary care, for better resolution and encouragement to good parenting practices in care and child development in home, this being the scope for further studies to increase specific policies and programs for home follow-up of vulnerable children and their families.
